# Characterization of five novel vasopressin V2 receptor mutants causing nephrogenic diabetes insipidus reveals a role of tolvaptan for M272R-V2R mutation

**DOI:** 10.1038/s41598-020-73089-x

**Published:** 2020-10-02

**Authors:** Federica Prosperi, Yoko Suzumoto, Pierluigi Marzuillo, Vincenzo Costanzo, Sabina Jelen, Anna Iervolino, Stefano Guarino, Angela La Manna, Emanuele Miraglia Del Giudice, Alessandra F. Perna, Miriam Zacchia, Emmanuelle Cordat, Giovambattista Capasso, Francesco Trepiccione

**Affiliations:** 1 Biogem S.c.a.r.l., Istituto Di Ricerche Genetiche “Gaetano Salvatore”, Ariano Irpino, Italy; 2 Department of Woman, Child and of General and Specialized Surgery, University of Campania “L. Vanvitelli”, Naples, Italy; 3 Department of Physiology, The University of Alberta, Edmonton, Canada; 4 Department of Translational Medical Sciences, University of Campania “L. Vanvitelli”, Naples, Italy

**Keywords:** Nephrons, Acid, base, fluid, electrolyte disorders

## Abstract

Nephrogenic diabetes insipidus (NDI) is a rare tubulopathy characterized by urinary concentration defect due to renal resistance to vasopressin. Loss-of-function mutations of vasopressin V2 receptor (V2R) gene (*AVPR2*) is the most common cause of the disease. We have identified five novel mutations L86P, R113Q, C192S, M272R, and W323_I324insR from NDI-affected patients. Functional characterization of these mutants revealed that R113Q and C192S were normally localized at the basolateral membrane of polarized Madin-Darby Canine Kidney (MDCK) cells and presented proper glycosylation maturation. On the other side, L86P, M272R, and W323_I324insR mutants were retained in endoplasmic reticulum and exhibited immature glycosylation and considerably reduced stability. All five mutants were resistant to administration of vasopressin analogues as evaluated by defective response in cAMP release. In order to rescue the function of the mutated V2R, we tested VX-809, sildenafil citrate, ibuprofen and tolvaptan in MDCK cells. Among these, tolvaptan was effective in rescuing the function of M272R mutation, by both allowing proper glycosylation maturation, membrane sorting and response to dDAVP. These results show an important proof of concept for the use of tolvaptan in patients affected by M272R mutation of V2R causing NDI.

## Introduction

Nephrogenic diabetes insipidus (NDI) is characterized by renal resistance to the hormone vasopressin (AVP). This leads to a defective water reabsorption along the renal collecting duct causing polyuria, hyposthenuria and polydipsia that can end up in life-threating dehydration and hypernatremia mainly in childhood^1^. NDI can be genetically determined or secondary mainly to hypokalemia, hypercalcemia/hypercalciuria^2^ or lithium salt exposure. All these conditions alter the V2 receptor (V2R)—aquaporin 2 (AQP2) axis, the main targets of water reabsorption in the principal cells of the collecting duct^3^. Recently, we and others showed experimentally induced integrin-b1 ablation leads to a dysfunction of the collecting duct and NDI because of an alteration of renal architecture^4–6^.

Congenital NDI is caused by loss-of-function mutations in genes encoding V2R or AQP2. Defects in V2R account for approximately 90% of congenital NDI with X-linked inheritance patterns, whereas AQP2 mutations can be transmitted in either autosomal dominant or recessive forms^7,8^. In the kidney, V2R is expressed at the basolateral membrane of principal cells of the distal nephron and in TAL cells^9,10^. The interaction with vasopressin in the principal cells triggers the activation of G proteins and a cascade of events mediated by cAMP and protein kinase A (PKA)^11^. In humans, the V2R protein encompasses 371 amino acids. About 300 mutations causing NDI have been identified^1^. The majority of them are in the coding region of the *AVPR2* gene and lead to single amino acid substitution. The remainder causes frame-shift or truncated proteins. Although some mutations impair ligand-binding or G-protein coupling, the majority of them alter proper V2R folding and impair its sorting to basolateral membrane by trapping the receptor in the endoplasmic reticulum (ER) quality control system^12^. Some pharmacological chaperones can rescue cell surface expression and function of ER-retained mutants of the V2R^13^. Pharmacological chaperones work as scaffold for the mutated V2R, stabilizing a near-native conformation of the receptor, thus allowing its release from the ER and sorting to basolateral membrane. In line with other diseases caused by loss-of-function mutation of GPCR as cystic fibrosis or other enzymes as Fabry disease^14,15^, a potential role of pharmacological chaperones also for NDI has been supported by in vitro evidences^16^ and a pilot clinical trial^17^.

In this study we characterized the phenotype of novel identified V2R mutations. We identified 3 V2R mutations retained in ER and 2 mutations presenting a proper sorting to basolateral membrane, but impaired cAMP generation by vasopressin analogues. In the attempt to rescue the disease by testing several potential pharmacological chaperones, we found that mutation M272R of V2R was responsive to tolvaptan both in term of maturation, membrane sorting and dDAVP response.

## Results

### Identification of 5 novel mutations of V2R

Five male patients were diagnosed with Nephrogenic Diabetes Insipidus according to clinical criteria^1^. The diagnosis was further confirmed by the identification of five novel NDI-causing mutations of *AVPR2* gene, namely L86P, R113Q, C192S, M272R, and W323_I324insR-V2R (Table 1). The onset of NDI differs among them. Patients bearing the mutations L86P, M272R, and W323_I324insR-V2R were diagnosed before 6 months of age, while the patient with C192S mutation after 18 months from the birth. Finally, the patient carrying R113Q mutation was diagnosed when he was 15 year-old, even though he declared that polydipsia was present since his infancy. All the patients were challenged with a dDAVP-stimulation test that confirmed an abnormal response (increase in urinary osmolality lower than 50% of the baseline), corroborating renal resistance to dDAVP.

**Table 1:** Clinical characterization of NDI patients with V2R mutation in this study.

Case	1	2	3	4	5
Name	L86P	M272R	W323_I324insR	R113Q	C192S
Type of mutation	Missense	Missense	In-frame insertion	Missense	Missense
Location^a^	TM2	TM6	TM7	ECL1	ECL2
Gender	M	M	M	M	M
Time to diagnosis	1 month	6 months	1.5 months	15 years	18 months
Serum Na^+^ (mM)	156	156	167	155	156
Serum Osm (mOsm/KgH_2_O)	316	320	345	315	317
U Osm pre dDAVP (mOsm/KgH_2_O)	229	65	82	140	120
U Osm post dDAVP (mOsm/KgH_2_O)	158	86	64	162	150

^a^TM, transmembrane; ECL, extracellular loop.

### Localization and maturation of five V2R variants in MDCK cells

**Figure 1:**
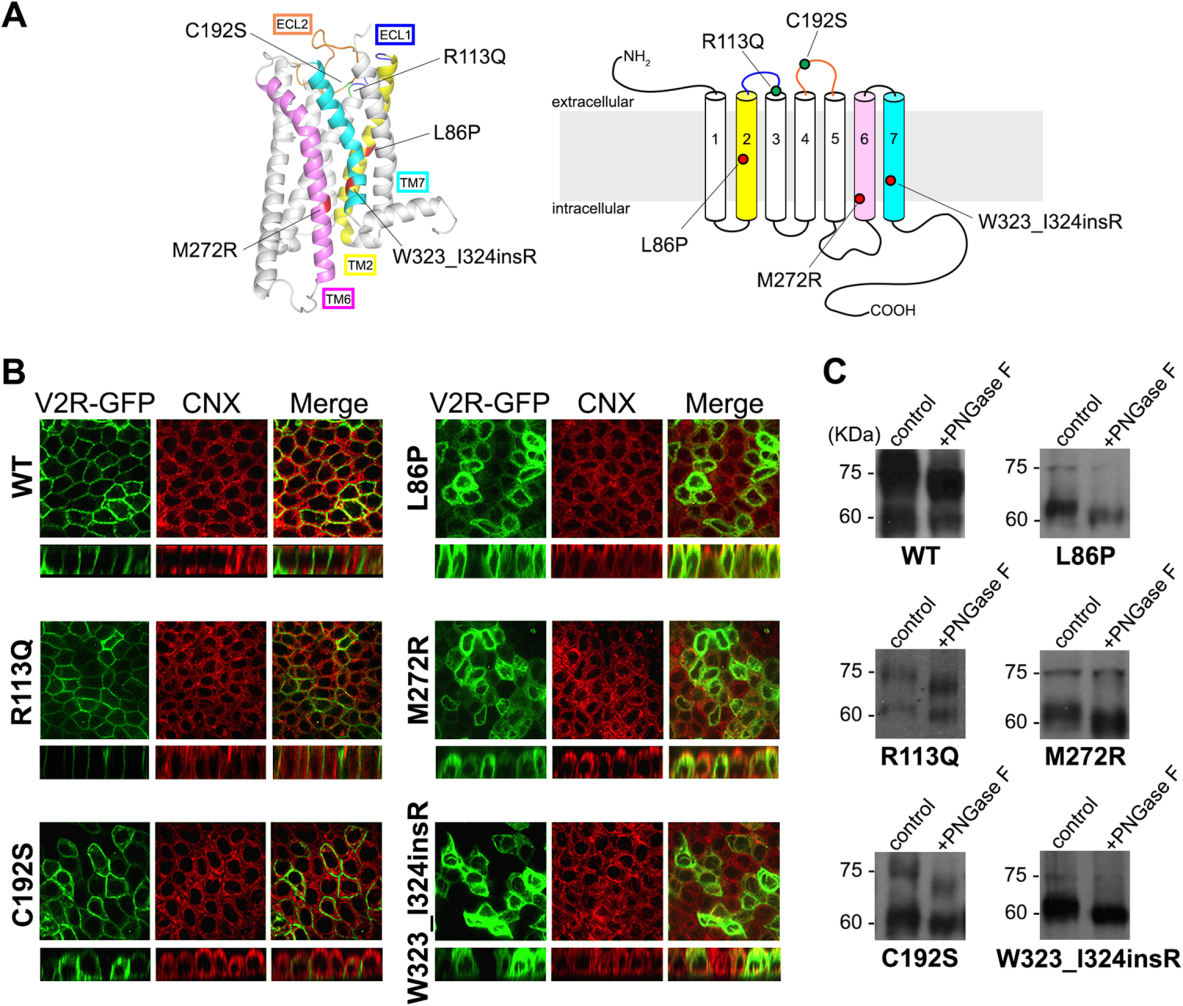
(**A**) Localization of five novel V2R mutations on the three-dimensional model (left) from GPCRdb database (https://gpcrdb.org) was illustrated using PyMol software and predicted topology diagram (right) of human V2R. ECL stands for Extracellular Loop; TM is for Transmembrane domain. (**B**) Cellular localization of NDI-causing V2R mutants. Representative pictures of MDCK cells expressing WT or mutated V2R-GFP (green) and calnexin (CNX, red), a marker of ER. WT, R113Q and C192S mutated V2R localized at basolateral membrane, while L86P, M272R and W323_I324insR co-localized with CNX. (**C**) Glycosylation maturation of NDI-causing V2R mutations evaluated by immunoblotting with or without PNGase F treatment. In WT, R113Q and C192S, both mature 75 kDa and immature 63 kDa V2R-GFP bands were down-shifted in size by treatment with PNGase F, suggesting the removal of N-linked oligosaccharides from V2R-GFP. L86P, M272R and W323_I324insR do not present a specific band at 75 kDa and the downshift occurs only in the 63 kDa band.

Five novel NDI-causing V2R mutations were further subjected to phenotypical characterization in vitro. Figure 1A shows the localization of the five mutations into a homology model of human V2R obtained from GPCRdb database (https://gpcrdb.org)^18^. L86P and M272R are single amino acid substitutions within transmembrane (TM) domain 2 and 6, respectively, based on the predicted topology model^19^. W323_I324insR mutation is located at TM7 including an insertion of arginine (R) residue between tryptophan at 323 and isoleucine at 324. Mutations R113Q and C192S map at the extracellular loops (ECL)1 and 2. In order to define the cellular localization of each single V2R mutation we generated five in-vitro models of the disease by transfecting Madin-Darby Canine Kidney (MDCK) type I cells with plasmids bearing the mutated V2R cDNA tagged in its carboxyl-terminus with GFP as previously shown^20^.

Immunocytochemistry of polarized MDCK cells confirms that WT-V2R is normally localized at basolateral membrane (Fig. 1B) as are R113Q and C192S-V2R mutants. Intracellular localization was observed for L86P, M272R and W323_I324insR mutations. These mutants colocalized with calnexin (CNX), a known marker of ER (Fig. 1B).

ER to Golgi complex transition is associated with protein maturation by adjunction of high-mannose oligosaccharides to asparagine residues (N-linked glycosylation)^21^. In order to evaluate proper protein maturation we performed immunoblotting of cell lysates with and without pre-treatment with peptide-N-glycosidase F (PNGase F) enzyme which specifically removes N-linked glycans from glycoproteins.

As previously reported, undigested WT-V2R showed a strong band of mature, complex-glycosylated protein, which corresponds to the band at 75 kDa (Fig. 1C). A second, weaker band around 60–63 kDa was also detected, which represents the immature, core-glycosylated receptors^20^. Digestion with PNGase F resulted in the cleavage of both complex-glycosylated 75 kDa WT-V2R and immature 63 kDa bands into O-glycosylated 67 kDa and non-glycosylated 60 kDa proteins, respectively. Glycosylation patterns of basolaterally expressed R113Q and C192S mutants were similar to that of WT-V2R (Fig. 1C). In contrast, under undigested condition, all ER-retained V2R mutants (L86P, M272R and W323_I324insR) were expressed as immature receptors of 63 kDa, accompanied by a minor band at 75 kDa which is presumably non-specific to V2R as it was unaffected by PNGase F digestion. The 63 kDa band was reduced to 60 kDa by cleavage with PNGase F, indicating that removal of high-mannose glycans resulted in the generation of non-glycosylated mutant receptors (Fig. 1C). These data suggest that all tested ER-retained mutants, but not the membrane expressed ones, lack complex-glycosylated oligosaccharides, supporting that they do not reach the Golgi.

### Stability of five V2R mutants

**Figure 2:**
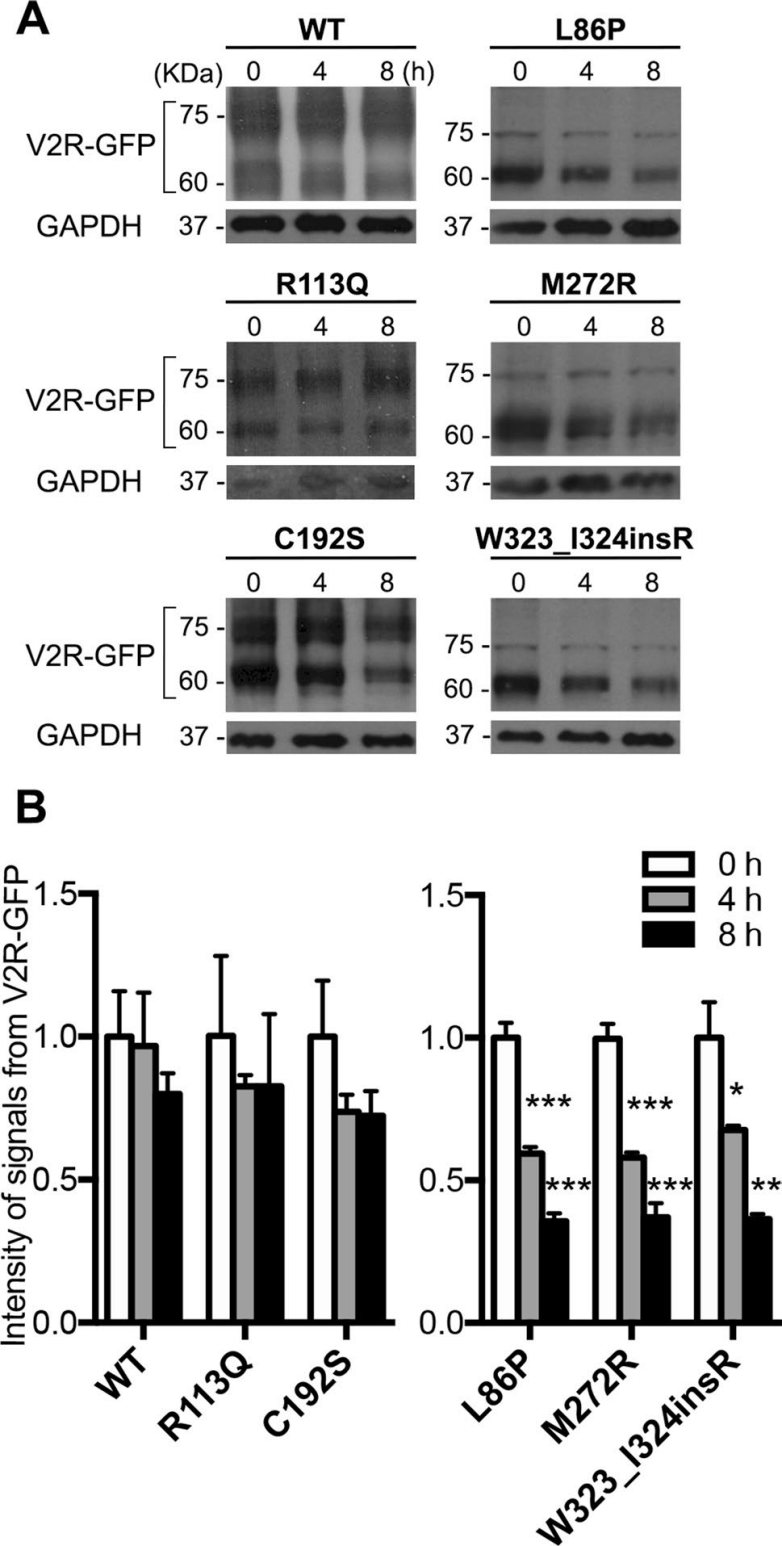
Stability of NDI-causing V2R mutants. (**A**) MDCK cells stably expressing WT, L86P, R113Q, C192S, M272R and W323_I324insR were treated with 100 μM cycloheximide (CHX) for 0, 4 and 8 h, followed by immunoblotting using anti-GFP antibody. (**B**) Protein abundance was normalized against endogenous GAPDH. Values are means ± SEM. (n = 3, *P < 0.05; **P < 0.01; ***P < 0.001; one-way ANOVA).

Gene mutations can affect proper protein folding and result in alteration of protein stability, mainly because of trapping the receptor in the ER quality control system. To test the protein stability, we performed a cycloheximide (CHX) chase assay. Protein synthesis in MDCK cells expressing WT-, Mock- and mutant V2R was blocked by incubation with CHX and residual protein abundance was evaluated at 0, 4 and 8 h time points by western blot (Fig. 2). WT-V2R abundance slightly decreased over time: 97% at 4 h and 80% at 8 h compared to baseline. The plasma membrane located mutants, R113Q and C192S-V2R behaved similarly to the WT, showing more than 70% compared to the baseline after 8 h. The ER retained mutants, instead, showed a severely reduced stability compared to WT. Indeed, L86P and M272R presented with less than 60% expression, and W323_I324insR was 68% compared to respective baseline already after 4 h of CHX incubation. Finally, after 8 h of CHX incubation, only 40% of residual expression was detectable for ER-retained mutants. The impairment in the stability of the ER-retained mutants suggests that the gene variation is associated with a misfolding and ER retention, followed by proteasomal/lysosomal degradation^20,22^.

### Evaluation of response to vasopressin analogues

**Figure 3:**
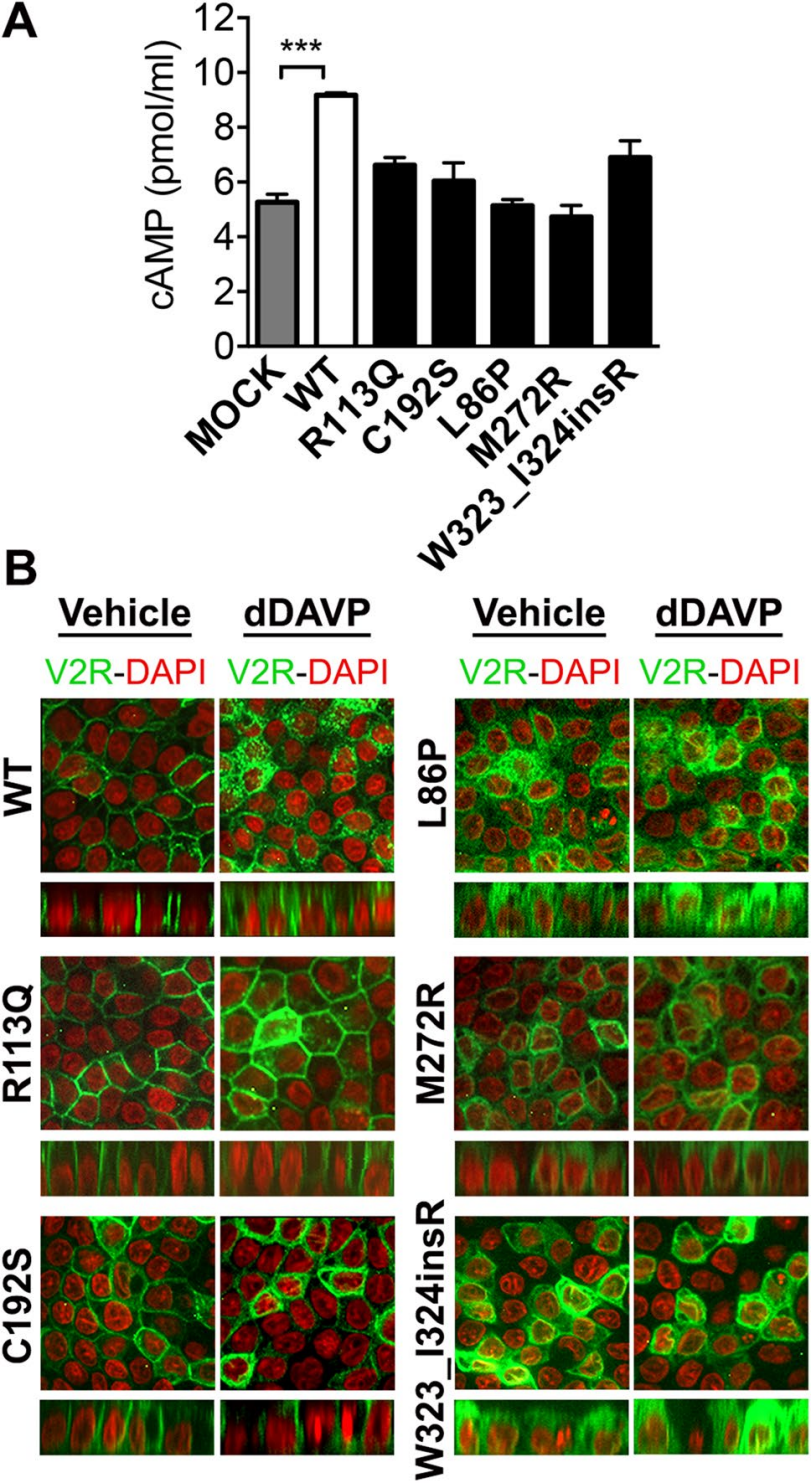
Response to dDAVP treatment. (**A**) cAMP generation in COS-7 cells expressing WT, Mock and NDI-causing V2R mutants. cAMP levels in COS-7 expressing WT and mutant V2R were compared with cAMP in Mock-expressing COS-7 cells. Values are means ± SEM (n = 3, ***P < 0.001, one-way ANOVA). (**B**) dDAVP-induced V2R internalization. Representative pictures of V2R-GFP (green) and nuclei stained by DAPI (red) revealed a proper resposnse to dDAVP only in cells expressing WT-GFP V2R.

To investigate whether the function of the mutated V2R was preserved, the ability to generate cAMP in response to a vasopressin analogue dDAVP has been evaluated. In order to exclude the effect derived from endogenously expressed V2R in MDCK cells^9^, we used COS-7 cells for the transient expression of WT-, Mock- and mutants V2R-GFP. COS-7 cells transiently expressing WT-V2R-GFP normally responded to 30 min dDAVP stimulation by generating a large cAMP release (Fig. 3A) compared to Mock. Almost no increase of cAMP signal was detected in L86P and M272R-transfected COS-7 cells. In R113Q, C192S, and W323_I324insR-transfected COS-7 cells, cAMP production was significantly lower than WT. Thus, these results suggest that ER-retained mutants are insensitive to dDAVP as they are intracellularly localized, while a severe dDAVP-response reduction can be seen in membrane located mutations.

Desensitization of GPCRs induced by their agonists is an important mechanism for the GPCRs-expressing cells to sustain regulated sensitivity to selective ligands^23^. This process includes internalization of the ligand-receptor from plasma membrane. Then receptors are either recycled to plasma membrane or subjected to lysosomal degradation^23^. It was previously demonstrated that dDAVP induces the internalization of V2R in dose- and time-dependent manners^9^. In order to assess the response of V2R variants to agonist binding, we treated polarized MDCK cells expressing WT-, Mock- or mutant V2R-GFP with 100 nM dDAVP for 1 h. In Fig. 3B, confocal laser scanning microscopy analysis showed that WT-V2R was translocated intracellularly after dDAVP incubation for 1 h. In contrast, dDAVP had no effect on the localization of all V2R mutants including the basolaterally-expressed R113Q and C192S mutants.

In order to test whether these two mutants respond to other vasopressin analogues, we exposed COS-7 cells transiently expressing WT, R113Q and C192S mutants with oxytocin or lysipressin. Both R113Q and C192S mutants did not improve cAMP release when exposed to oxytocin, lysipressin or dDAVP (Fig. S1).

### Evaluation of response to pharmacological chaperones

Pharmacological chaperones are molecules that serve as scaffold for misfolded proteins and contribute to reach the proper folding^13–15^. These molecules have been specifically used for ER-retained mutants of GPCRs. In particular, VX-809, sildenafil citrate and ibuprofen have been successfully used to rescue the misfolded F508del-CFTR mutant^24–28^ causing a common form of cystic fibrosis. We applied these pharmacological chaperones on the ER-retained V2R mutants that we identified (L86P, M272R and W323_I324insR). However, VX-809, sildenafil citrate and ibuprofen were not effective in improving maturation or rescuing them to the basolateral membrane (Figs. S2 and S3).

**Figure 4:**
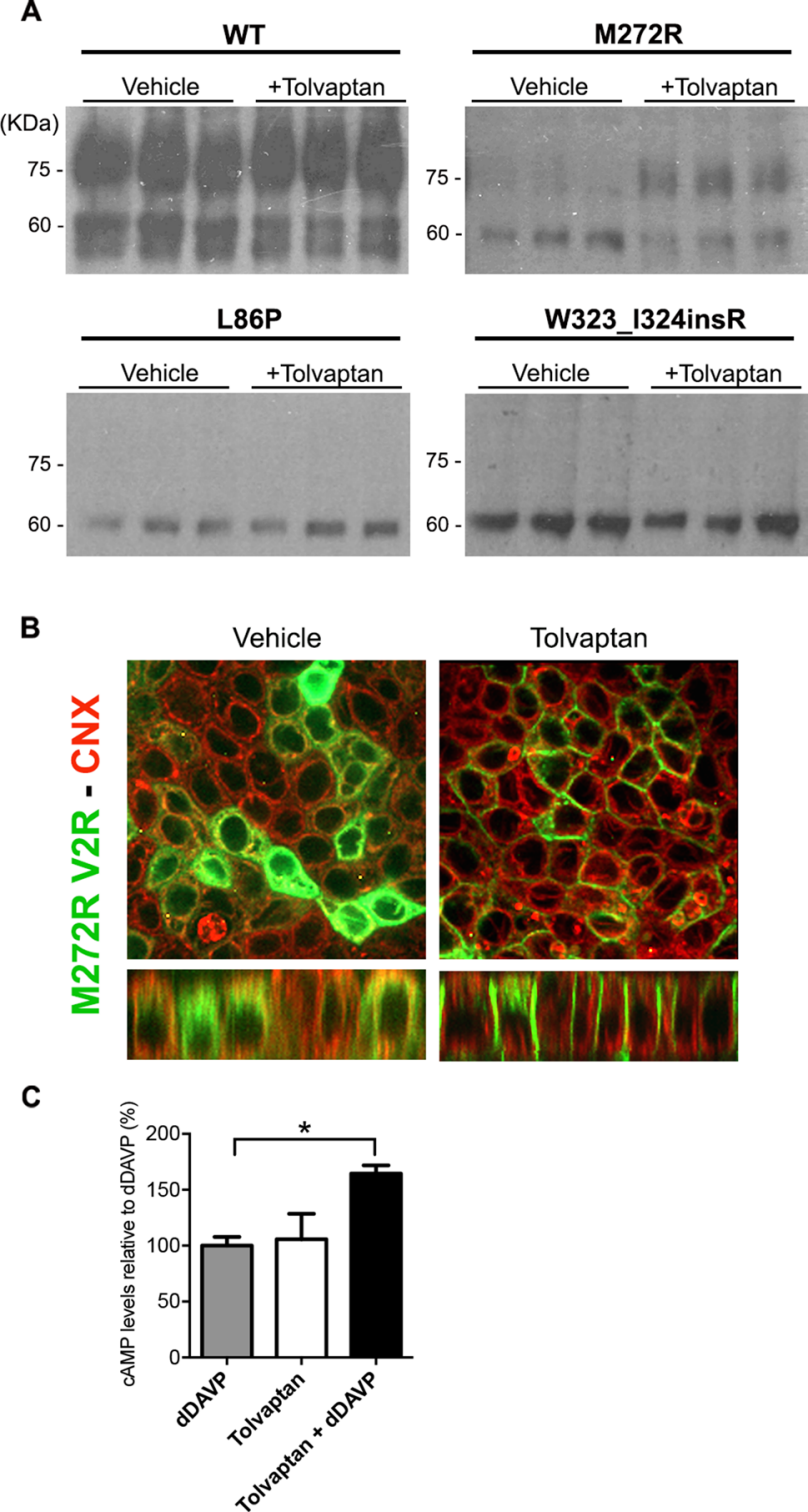
Rescue of ER-retained V2R mutants by tolvaptan. (**A**) Maturation of WT, L86P, M272R and W323_I324insR-V2R after treatment with 1 μM tolvaptan for 16 h. Cells were lysed and analysed by immunoblotting using anti-GFP antibody. Tolvaptan was effective in ameliorating the glycosylation maturation of M272R mutants. (**B**) Representative pictures of M272R-V2R-GFP in polarized MDCK cells showed restoration of basolateral membrane expression of M272R after treatment with tolvaptan. (**C**) Generation of cAMP in MDCK cells stably expressing M272R variant treated with or without tolvaptan and following dDAVP stimulation. M272-expressing MDCK cells treated with tolvaptan showed significantly increased level of cAMP after stimulation with dDAVP. Values are means ± SEM (n = 4, *P < 0.05; one-way ANOVA).

A membrane permeable V2R antagonist named tolvaptan, previously known as OPC4, was shown to rescue several V2R NDI-causing mutants^29–31^. Treatment with tolvaptan improved the appearance of the complex-glycosylated band at 75 kDa in M272R mutant associated to a decrease of the immature 60–63 kDa band. No effect was found in the mutants L86P and W323_I324insR. (Fig. 4A). This may imply that M272R-V2R mutant translocates to the Golgi complex where complex-glycosylation occurs. Confocal laser scanning microscopy analysis corroborated this point. Treatment with 1 µM tolvaptan for 16 h allowed the M272R-V2R translocation to basolateral membrane (Fig. 4B). Finally, the addition of dDAVP to tolvaptan-pre-treated M272R-V2R cells increased the cAMP levels by about 1.5-fold compared to non-tolvaptan pre-treatment (Fig. 4C). V2R-mutants L86P and W323_I324insR did not respond to tolvaptan. By performing a docking analysis between tolvaptan and the M272R-V2R we could predict in the TM1 and TM7 a novel cluster of binding sites not identified in the wild type V2R (Fig. S4). In conclusion, tolvaptan works as a pharmacological chaperone for M272R-V2R mutant rescuing its ER retention and triggering its full glycosylation maturation, membrane expression and dDAVP response.

## Discussion

In this study we have characterized the function of five newly identified V2R mutants. Among these R113Q and C192S-V2R were properly localized at basolateral membrane, presenting no significant alteration in glycosylation maturation and stability compared to WT-V2R. However, they were unresponsive to dDAVP and other vasopressin analogues, oxytocin and lysipressin. Both R113Q and C192S V2R are localized in two extracellular domains, ECL1 and ECL2, respectively.

Importantly, C192 at the second extracellular loop (ECL2) is thought to constitute a covalent disulphide bond with another cysteine residue at 112 (C112) in the ECL1^32^. This disulphide bond formed by two conserved cysteine residues plays an essential role on the correct folding of ECL1 and 2, thereby establishing a proper conformation of the putative ligand binding domain of V2R. Therefore, C192S mutation could alter V2R function by preventing ligand binding to the same extent as other mutants targeting the amino acids at position 192 and 112, namely C112S, C112A and C192A^32^. This could explain the selective resistance of C192S mutant to the vasopressin analogues, even in the presence of normal maturation and membrane expression.

Meanwhile, in contrast to our result showing the basolateral membrane localization of C192S in polarized MDCK cells, Schülein et al*.* found an intracellular localization of C192S in transiently transfected HEK293 cells^32^, likely due to absence of polarization and/or different transfection technique (stable for MDCK vs. transient for HEK293 transfection)^33^.

Alterations of the putative ligand binding domain of V2R could potentially occur also with the R113Q-V2R mutation. The exchange of arginine for glutamine at position 113 could have an impact on ligand binding affinity through the elimination of a positive charge at this site. Indeed, Kojro et al. identified R113 in the hormone binding domain by photo-affinity labelling of bovine V2R with a tritium-labelled photoreactive vasopressin agonist^34,35^. On this line the mutation R113W, replacing arginine with tryptophan, showed a lower affinity for AVP and reduced coupling to G_s_/adenylyl cyclase system^36^. Saturation mutagenesis study at this amino acid residue will be required to fully understand the role of R113 on ligand binding.

On the other hand, L86P, M272R and W323_I324insR-V2R mutants exhibited ER-retention and immature glycosylation with considerably reduced stability. These ER-retained mutants were unable to respond to dDAVP. All mutations site were localized within the transmembrane domain.

CFTR correctors including Sildenafil citrate, ibuprofen, VX-809 were effective in rescuing some CFTR mutations^24–28^, however, they had no effect for the V2R mutants that we studied. Sildenafil was reported to improve the water retention in a patient affected by deletion of Arg-247 to Gly-250 of V2R^37^, but this effect seems likely due to a non-chaperone like effect of the drug.

Among more than 300 NDI-associated V2R mutations identified to date, several point mutations include a single amino acid substitution with proline occurring within the transmembrane domains. Due to the lack of amide group, proline is unable to form conventional intra-helical hydrogen bonds^38^ and this could affect the transmembrane α-helices folding (Fig. S5). Indeed, several mutants carrying a substitution into a proline (L44P, L59P, L219P, L282P and L292P) including our L86P^29,39–42^ exhibited intracellular localization.

The W323_I324insR mutation of V2R is located at the end of TM7 extending to the cytoplasmic COOH terminal tail. The amino acid region 321 – 325 of human V2R (N321, P322, W323, I324 and Y325) constitutes a so-called NPxxY motif, which is highly conserved among GPCRs. The NPxxY motif plays an essential role on the receptor downregulation via clathrin-mediated endocytosis in several GPCRs including V2R^43^. In addition, in the family of beta 2-adrenergic receptors, it was found fundamental also for G-protein signalling^44,45^. The identified W323_I324insR mutation of V2R alters the conserved NPxxY motif by inserting an arginine at position 324 and replacing tyrosine with isoleucine at position 325. Besides conceivable functional defects due to its involvement in the NPxxY motif, this mutation could also contribute to V2R misfolding. Indeed, homology model of W323_I324insR indicates the disruption of alpha-helix of TM7 due to the insertion of arginine at position 324 (Fig. S5). Although the mutation Y325F of V2R was reported to have no effect on the basolateral localization of the receptor^43^, this may be due to the synonym substitution between two aromatic amino acids. In contrast, W323_I324insR includes the replacement of an aromatic with an aliphatic amino acid at position 325 severely altering the normal protein folding.

Finally, M272R mutation of V2R is located at the edge of TM6. Another variant, M272K, at same amino acid position was previously reported to cause NDI, with poor phenotypical characterization^41^. Both M272R and M272K mutations include an exchange from hydrophobic to positively charged residue, being potentially detrimental for the conformation of the TM domain. In contrast to ER-retained L86P and W323_I324insR mutations, M272R-V2R phenotype was rescued by tolvaptan both in terms of protein maturation, localization and response to dDAVP.

Previously tolvaptan was also shown to rescue L44P, I130F, S167T, Y128S and S333del-V2R variants^29–31^. Among them, Y128S and S333del mutations were identified from patients with partial NDI^30^, while our identified mutation responsive to tolvaptan, namely M272R together with mutation I130F^46^, led to a severe urinary concentration defect (Table 1, Table S1). By modelling these mutations, it seems crucial for the chaperone activity of tolvaptan to act on mutations that preserve the integrity of the H-bonds in the TM alpha-helices. Indeed, this is true for M272R, Y128S, S333del and I130F mutations, but not for L86P (Fig. S5, Table S1), suggesting a potential role of other mechanisms.

Furthermore, it is likely that the extent of cell surface restoration by pharmacological chaperones is not only depending on the position of mutated amino acid, but also on the amino acid exchange which determines the severity of mutation on protein folding. Indeed, even though S167T mutation was successfully rescued by tolvaptan and by two other pharmacological chaperones including OPC31260 (OPC3) and SR121463B (SR1), the mutation at the same position S167L was not rescued by either of these antagonists^29^. The substitution of serine into threonine at 167 has only a slight impact on the interaction with adjacent residue V121, whereas amino acid exchange to leucine causes a large disturbance on 3D structure of V2R, which cannot be rescued by antagonists^47^.

Tolvaptan as well as other non-peptide pharmacological chaperones such as OPC21268 (OPC2), OPC3, and SR directly bind to misfolded V2R retained in ER, and assist proper folding of mutant receptors by enhancing their conformational stability^47^. In silico docking of AVP and these pharmacological chaperones on V2R model structure showed a partial overlapping between binding domains of AVP and pharmacological chaperones^48^. However, pharmacological chaperones penetrate deeper into transmembrane region whereas AVP binds mainly at the extracellular surface of V2R. Extended interaction between pharmacological chaperones and transmembrane domains of V2R presumably provides an essential driving force for the proper folding of mutant receptors^47^.

Notably, the rescue activity of pharmacological chaperones are depending on their chemical properties, as docking analysis between V2R and non-peptide pharmacological chaperones pointed out different residues directly in contact with these antagonists^48^. This could also in part explain the different effects of pharmacological chaperones on various V2R mutants^16^. In this regard, it would be valuable to test other non-peptide pharmacological chaperones for ER-retained L86P and W323_I324insR variants which were not rescued by tolvaptan.

In conclusion, we have functionally characterized in vitro 5 mutations of the V2R. Two mutants (R113Q and C192S) were expressed at the basolateral membrane and the other 3 were retained in the ER (L86P, M272R and W323_I324insR). We have tested several pharmacological chaperones in order to rescue the NDI-leading phenotype and demonstrated a successful use of tolvaptan for rescuing the M272R-V2R mutant. This contributes to the backbone of evidence for a pharmacological chaperone approach to X-linked NDI.

## Methods

### Reagents

All the procedures were performed according to the Italian Ministry of Health decree nr 100/2006 and later decree 26/2014. All reagents for cell culture including fetal bovine serum (FBS), l-Glutamine, Penicillin–Streptomycin, nonessential amino acids and Geneticin (G418 sulphate) were purchased from Euroclone (Pero, MI, Italy) except for Dulbecco’s modified Eagle’s medium (DMEM) from Lonza (Basel, Switzerland). Vasopressin analogue desmopressin (dDAVP) was obtained from Kedrion Biopharma (Barga, LU, Italy). The V2R antagonist tolvaptan, F508del-CFTR correctors sildenafil citrate, ibuprofen, VX-809 and synthetic vasopressin analogues oxytocin and lysipressin were supplied by MedChem Express (Monmouth Junction, NJ, USA).

### Generation of expression constructs

The plasmid DNA pEGFP-N1-V2R encoding WT V2R COOH terminally-tagged with the green fluorescent protein (GFP) was used. Each mutation was introduced into human V2R cDNA sequence by QuikChange Site-Directed Mutagenesis Kit (Stratagene, Heidelberg, Germany) using pEGFP-N1-V2R as a template. The primers used are reported in the Supplementary Table S2. After double-digestion with EcoRI and BamHI restriction enzymes (NEB BioLabs, Ipswich, MA, USA), mutation-containing DNA fragments were cloned into corresponding site in WT-V2R-GFP. Sequence analysis of selected clone was performed to verify the presence of the desired mutations.

### MDCK stable cell line generation

Madin-Darby Canine Kidney (MDCK) cells were grown in DMEM (Lonza, Basel, Switzerland) supplemented with 5% fetal bovine serum, 2% l-Glutamine, 1% Pen/strep and 1% nonessential amino acids (Euroclone, Pero, MI, Italy) at 37 °C with 5.0% CO_2_. Expression constructs encoding WT- and mutant V2R-GFP, and empty pEGFP-N1 vector (Mock-transfection) were linearized by digestion with ApalI (NEB BioLabs, Ipswich, MA, USA) and transfection in MDCK cells was performed using Lipofectamine 2000 (Thermo Fisher Scientific, Waltham, MA, USA). Selections for positive clones stably expressing V2R proteins were carried out using 600 µg/ml G418 Sulphate (Geneticin, Euroclone, Pero, MI, Italy) and single clones of each mutants were isolated by fluorescence-driven screening.

### Immunocytochemistry

The protocol for immunohistochemistry was adapted from previous study as following^49^. MDCK cells stably expressing WT-, Mock- and mutant V2R-GFP were seeded on semi-permeable Costar filters (Corning, New York, NY, USA) at a density of 0.3 × 10^6^ cells/cm^2^ and grown for 5 days to induce polarization. The cells were fixed with 4% paraformaldehyde (PFA), washed with PBS and permeabilized for 15 min with a permeabilization solution (0.1% BSA, 0.3% Triton X-100 in PBS). The cells were then incubated with 50 mM NH_4_Cl in PBS for 15 min, and washed with PBS. Blocking with 16% Normal Goat Serum in PBS with 0.3% Triton X-100 was performed for 30 min followed by incubation with primary antibody rabbit anti-Calnexin (CNX) antibody^50^ in 1:500 dilution at 4 °C overnight. After washing, incubation with a secondary antibody goat anti-rabbit Cyanine3-conjugated (1:800 dilution, A10520, Invitrogen, Waltham, MA, USA) was performed for 45 min at room temperature. The Costar filters were washed in PBS and mounted on the slide glasses using fluorescent mounting medium (Dako, Carpinteria, CA, USA). Zeiss spinning disk Axio Observer Z1 confocal microscope (Zeiss, Oberkochen, Germany) was used for the acquisition of images.

### In vitro experiments with vasopressin analogue and pharmacological chaperones

In order to assess the response to vasopressin analogue dDAVP, MDCK cells stably expressing WT and V2R variants were grown to induce polarity for 5 days on Costar filters. After overnight serum starvation they were treated with 100 nM dDAVP for 1 h in serum-free media. Nuclei were stained by incubation with DAPI (1:2000 dilution) for 1 min. Treatment with pharmacological chaperones was performed in MDCK cells stably expressing WT-, L86P, M272R and W323_I324insR-V2R-GFP. After reaching 80% confluence, cells were incubated with serum-free media containing 1 µM of tolvaptan for 16 h or CFTR correctors at 1 µM for 24 h. Cells were rinsed with PBS twice prior to incubation with 100 nM dDAVP in serum-free media for 1 h. Immunocytochemistry and image acquisition were performed as described above. All compounds used were dissolved in dimethyl sulfoxide (DMSO, Sigma-Aldrich, Milan, Italy) as 10 mM stock solutions and diluted in culture medium as indicated.

### Immunoblotting

MDCK cells stably expressing WT or mutant V2R-GFP were grown on six-well plates until 80% confluence, collected and lysed with lysis buffer containing 1% NP-40 (Sigma-Aldorich, Milan, Italy), protease (Complete Protease Inhibitor Cocktail, Santa Cruz, USA) and phosphatase inhibitor cocktail (PhosSTOP, Roche, Monza, Italy). The lysates were centrifuged at 4000 ×*g* for 20 min, 4 °C, and supernatant was collected. Protein concentration was quantified using Pierce BCA Protein Assay Kit (Thermo Fisher Scientific, Waltham, MA, USA). Immunoblotting was performed as previously reported^51^. Briefly, twenty microgram of total protein samples mixed with 5 × Laemmli Buffer were separated by SDS-PAGE using 10% polyacrylamide gel, transferred on the nitrocellulose membrane, and incubated with primary antibodies at 4 °C overnight. Rabbit anti-GFP serum (1:7000 dilution) or rabbit anti-GAPDH (1:20,000 dilution, GTX100118, GeneTex, Hsinchu City, Taiwan) was used to label V2R-GFP or GAPDH for normalization, respectively. Subsequently incubation with secondary antibody anti-rabbit HRP-conjugated (1:2000 dilution, NA934V, GE Healthcare, Little Chalfont, UK) was performed for 1 h at room temperature, and nitrocellulose membranes were developed by Pierce ECL Western Blotting substrate (Thermo Fisher Scientific, Waltham, MA, USA). ImageJ software was used to quantify single band intensity. Data from three independent experiments were used for the quantification.

PNGase F (NEB BioLabs, Ipswich, MA, USA) was used to remove the N-linked glycans from proteins expressed in MDCK cells, according to the manufacturer’s instructions. In brief, twenty microgram of protein sample was mixed with 10× Glycoprotein Denaturing Buffer (supplied with kit) and denatured by heat reaction at 100 °C for 10 min. After cooling down, the protein sample was digested with PNGase F in the presence of 1× GlycoBuffer and 1% NP-40 at 37 °C for 1 h. The glycosylation states of WT- and mutants V2R-GFP were then analyzed by immunoblotting as already described.

### Cycloheximide chase assay

MDCK cells stably expressing WT- and mutant V2R-GFP were grown to 80% confluence, and incubated with the growth medium containing 100 μM cycloheximide (CHX, Sigma-Aldrich, Milan, Italy) for 0, 4, or 8 h. The cells were subsequently lysed with the solution containing 1% NP-40, protease (Complete Protease Inhibitor Cocktail, Santa Cruz, USA) and phosphatase inhibitor cocktail (PhosSTOP, Roche, Monza, Italy), and immunoblots were performed as described above. Protein concentration was quantified using Pierce BCA Protein Assay Kit (Thermo Fisher Scientific, Waltham, MA, USA).

### Intracellular cAMP measurement

In order to measure the cAMP production upon stimulation with dDAVP or oxytocin or lysipressin, COS-7 cells were used for transient expression of WT-, Mock- and mutant V2R-GFP. COS-7 cells were maintained in DMEM supplemented with 10% fetal bovine serum, 1% l-Glutamine, and 1% Penicillin–Streptomycin at 37 °C with 5.0% CO_2_. COS-7 cells grown on 6-well plate were transiently transfected with Lipofectamine 2000 using 1 μg of expression construct. After 48 h, cells were treated with 100 nM dDAVP for 30 min in serum free media and lysed with 0.1 M HCl. cAMP was measured using a direct cAMP ELISA kit (Enzo life sciences, Farmingdale, NY, USA) following the manufacturer’s instructions. Absorbance at 405 nm was measured with Absorbance Microplate Reader ELx800 (BioTek, Winooski, VT, USA). Measurements were performed in triplicate.

### Structural model of V2R

Homology model of human V2R in an inactive state was obtained from GPCRdb database (https://gpcrdb.org)^18^. The three-dimensional structure of L86P, M272R, W323_I324insR, I130F, Y128S and S333del mutants were generated by Swiss-Model protein structure homology-modelling server (https://swissmodel.expasy.org) using the homology model of WT human V2R as template. The visualization of V2R model structure was performed using PyMol software.

### Urinary osmolality measurement

Urinary osmolality was measured by freezing point depression osmometer (Model 3320, Advanced Instruments, Inc., MA, United States) as previously^52^.

### Ethics

Informed consent was obtained from all participants and/or their legal guardians for the anonymous publication of the data.

### Statistics

Values are represented as mean ± standard error (SEM). Data were analysed by one-way ANOVA test. P-value < 0.05 was considered to be significant. All the experiments were performed in triplicate.

## Supplementary information

Supplementary Information.
