# Supplementary material for: Characterization of five novel vasopressin V2 receptor mutants causing nephrogenic diabetes insipidus reveals a role of tolvaptan for M272R-V2R mutation
Source: Sci Rep. 2020 Oct 2;10:16383. doi: 10.1038/s41598-020-73089-x (PMC7532466; doi:10.1038/s41598-020-73089-x)

Supplementary Information

Characterization of five novel vasopressin V2 receptor mutants causing nephrogenic diabetes insipidus reveals a role of tolvaptan for M272R-V2R mutation.

Federica Prosperi, Yoko Suzumoto, Pierluigi Marzuillo, Vincenzo Costanzo, Sabina Jelen, Anna Iervolino, Stefano Guarino, Angela La Manna, Emanuele Miraglia Del Giudice, Alessandra F. Perna, Miriam Zacchia, Emmanuelle Cordat, Giovambattista Capasso, Francesco Trepiccione.

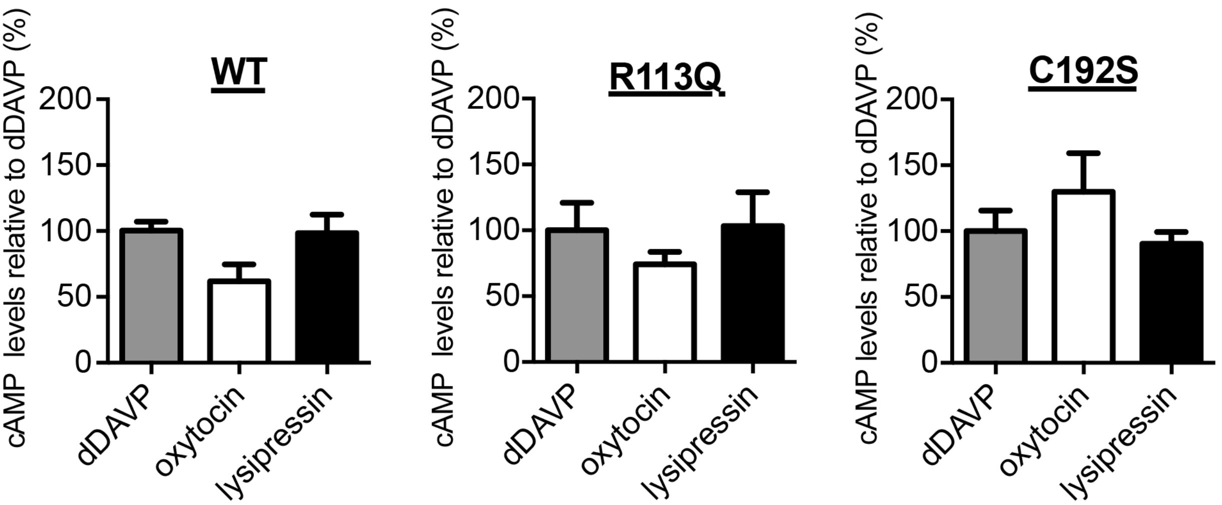

Supplementary Figure S1. Comparison of AVP analogues for stimulation of cAMP generation in basolaterally-expressed V2R. COS-7 cells transiently expressing WT, R113Q or C192S-V2R-GFP were treated with 100 nM dDAVP, oxytocin or lysipressin for 1h and lysed to measure the cAMP generation as described in Methods section. cAMP levels are represented as percentage with respect to cAMP level in cells treated with dDAVP. Values are means ± SEM (n=3-4 per each condition, one-way ANOVA).

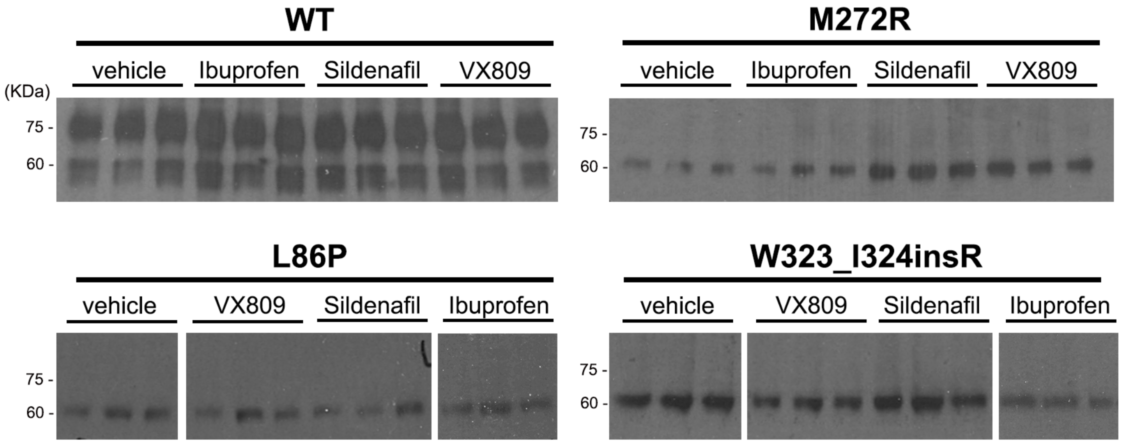

Supplementary Figure S2. Maturation of ER-retained V2R mutants treated with F508del-CFTR correctors. MDCK cells expressing WT, L86P, M272R or W323_I324insR-V2R-GFP were treated with 1 µM of CFTR correctors (VX-809, sildenafil or ibuprofen) for 24 h. Cells were lysed and analysed by immunoblotting using anti-GFP antibody.

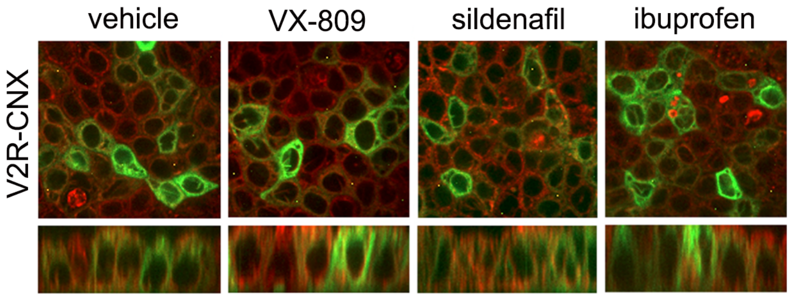

Supplementary Figure S3. Localization of M272R-V2R-GFP in polarized MDCK cells treated with VX-809, sildenafil and ibuprofen. Representative confocal laser scanning microscopy images of M272R-V2R-GFP (green) and ER marker CNX (red).

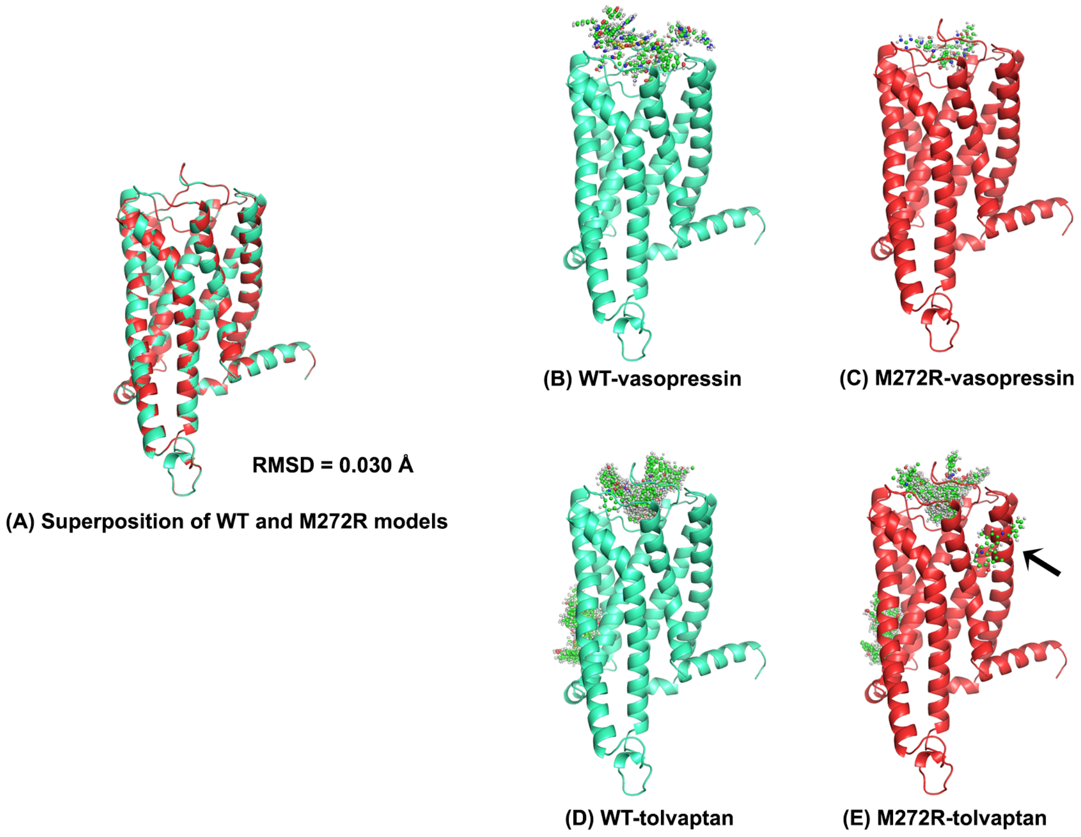

Supplementary Figure S4. Docking analyses for binding of vasopressin or tolvaptan to WT and M272R-V2R. Panel A represents the superposition of homology models of WT and M272R-V2R used in the docking. The root-mean-square deviation (RMSD) was calculated as 0.030 Å, indicating that two models possess similar 3D structures. Molecular dockings of vasopressin with WT receptor (B), or M272R-mutated receptor (C) or tolvaptan with WT receptor (D), or M272R-mutated receptor (E) was performed by SwissDock server (http://www.swissdock.ch). The parameters for docking were set as 'Accurate' for docking type. The 3D structure of vasopressin was obtained from the crystal structure of trypsin in complex with vasopressin (PDB: 1YF4) as ligand input. Tolvaptan was extracted from ZINC database (http://zinc.docking.org, ZINC538658). Clusters of predicted ligand bindings are shown as dots. For dockings of vasopressin binding (B and C), only the predicted clusters located at extracellular domains are shown. Comparison of vasopressin-binding between WT and M272R demonstrated a reduced number of predicted clusters for M272R (3 clusters for WT, 1 cluster for M272R), implying a possibly lower binding affinity of vasopressin for M272R. Finally, the docking analysis of tolvaptan-binding to M272R revealed an additional putative binding site in the space between TM1 and TM7 as indicated by black arrow (E), which was not predicted in the WT-receptor (D). Structural figures were prepared using PyMol software.

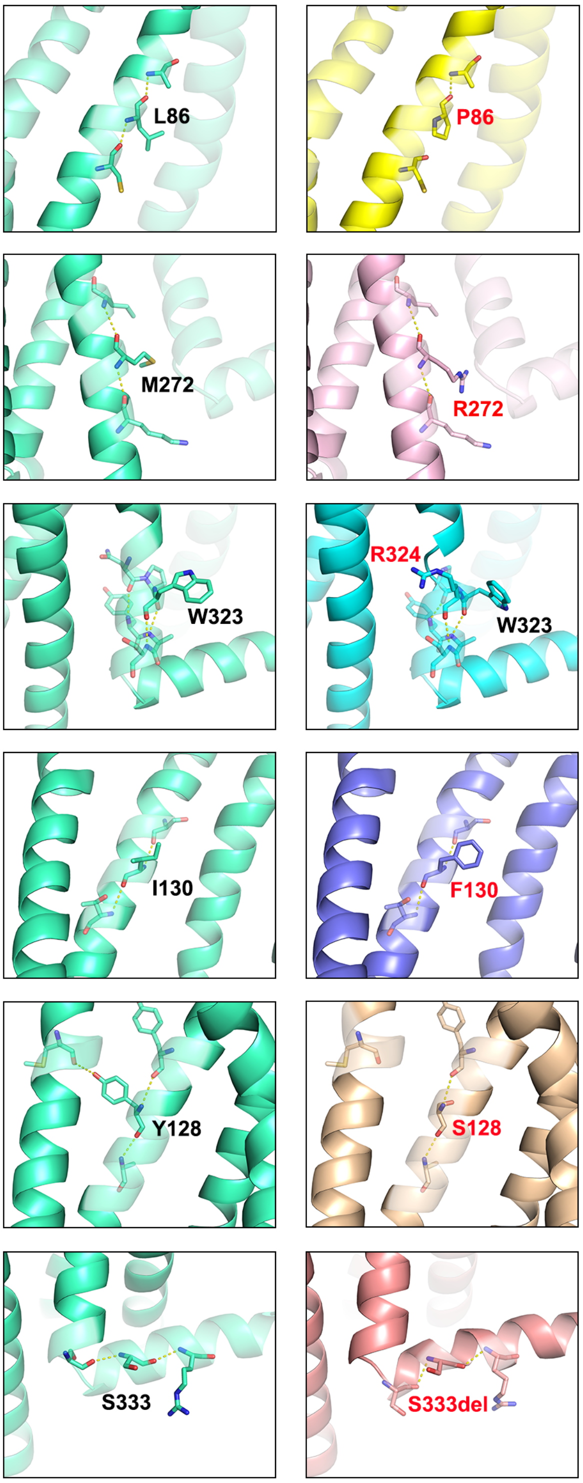

Supplementary Figure S5. Simulation of the effects of three ER-retained V2R mutations in this study (L86P, M272R and W323_I324insR) and other tolvaptan responsive V2R mutations (I130F, Y128S and S333del). Structural figures were prepared using PyMol software. Comparison between WT (left panels) and mutants (right panels) are represented in cartoon. The yellow dotted lines represent H-bonds. Note that all mutations except S333del are located in transmembrane alpha-helices.

Supplementary Table S1. Summary of analyses on protein structure and phenotype of V2R mutations in this study and related mutants

mutation	Structural alteration[a]	Phenotype[b]	Rescue by tolvaptan	Reference
L86P	severe	severe	No	This study
M272R	mild	severe	Yes	This study
W323_I324insR	severe	severe	No	This study
I130F	mild	severe	Yes	29, 46
Y128S	mild	mild	Yes	30
S333del	mild	mild	Yes	30

[a] Structural alteration was assessed by homology models of mutants shown in Supplementary Fig S5. Mutations potentially causing disturbance of transmembrane alpha-helices are assigned as severe, whereas mutations not associated with disruption of TM alpha-helices are described as mild.

[b] Severity of phenotypes were assessed by urine concentration ability evaluated by urinary osmolality of patients.

Supplementary Table S2. Sequence of primer pairs used for site-directed mutagenesis in this study

Primer	Sequence
hV2R L86P Fw	GTGCCTGGCCGACCCGGCCGTGGCTCTGTTC
hV2R L86P Rev	GAACAGAGCCACGGCCGGGTCGGCCAGGCAC
hV2R R113Q Fw	CAGATGCCCTGTGTCAGGCCGTGAAGTATCTG
hV2R R113Q Rev	CAGATACTTCACGGCCTGACACAGGGCATCTG
hV2R C192S Fw	GCGGGGTCACTGACAGCTGGGCCTGCTTTG
hV2R C192S Rev	CAAAGCAGGCCCAGCTGTCAGTGACCCCGC
hV2R M272R Fw	CCAAGACTGTGAGGAGGACGCTAGTGATTG
hV2R M272R Rev	CAATCACTAGCGTCCTCCTCACAGTCTTGG
hV2R W323_I324insR Fw	GCACCAACCCCTGGAGGATCTATGCATCTTTC
hV2R W323_I324insR Rev	GAAAGATGCATAGATCCTCCAGGGGTTGGTGC

Supplementary Figure S6. Full-length blots presented in Fig. 1C, 2A, 4A and Supplementary Fig.S2

Fig. 1C

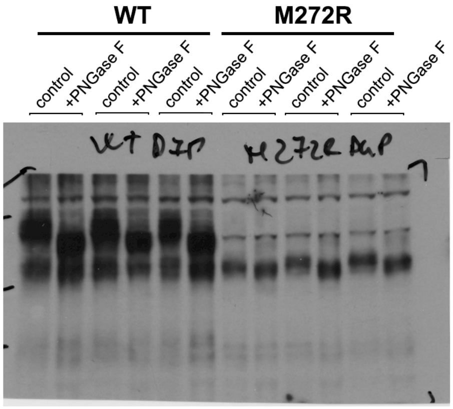

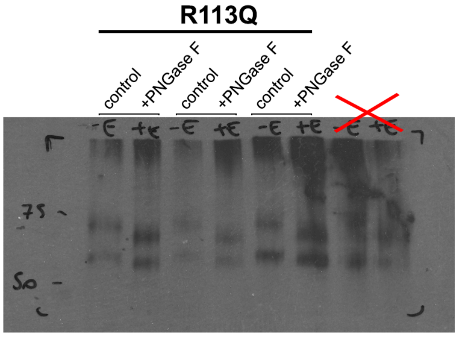

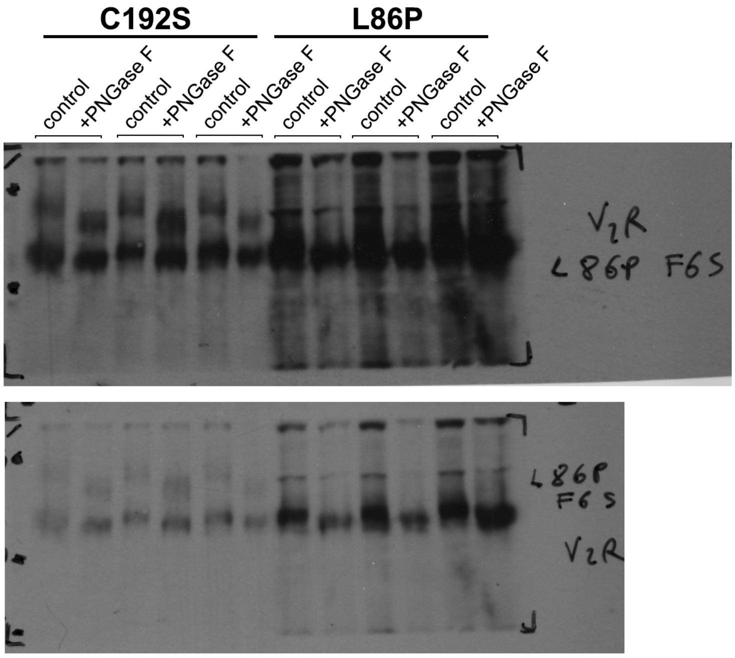

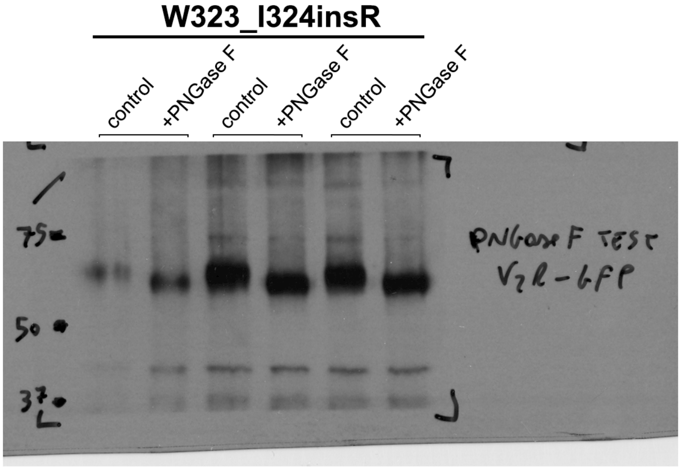

Fig. 2A

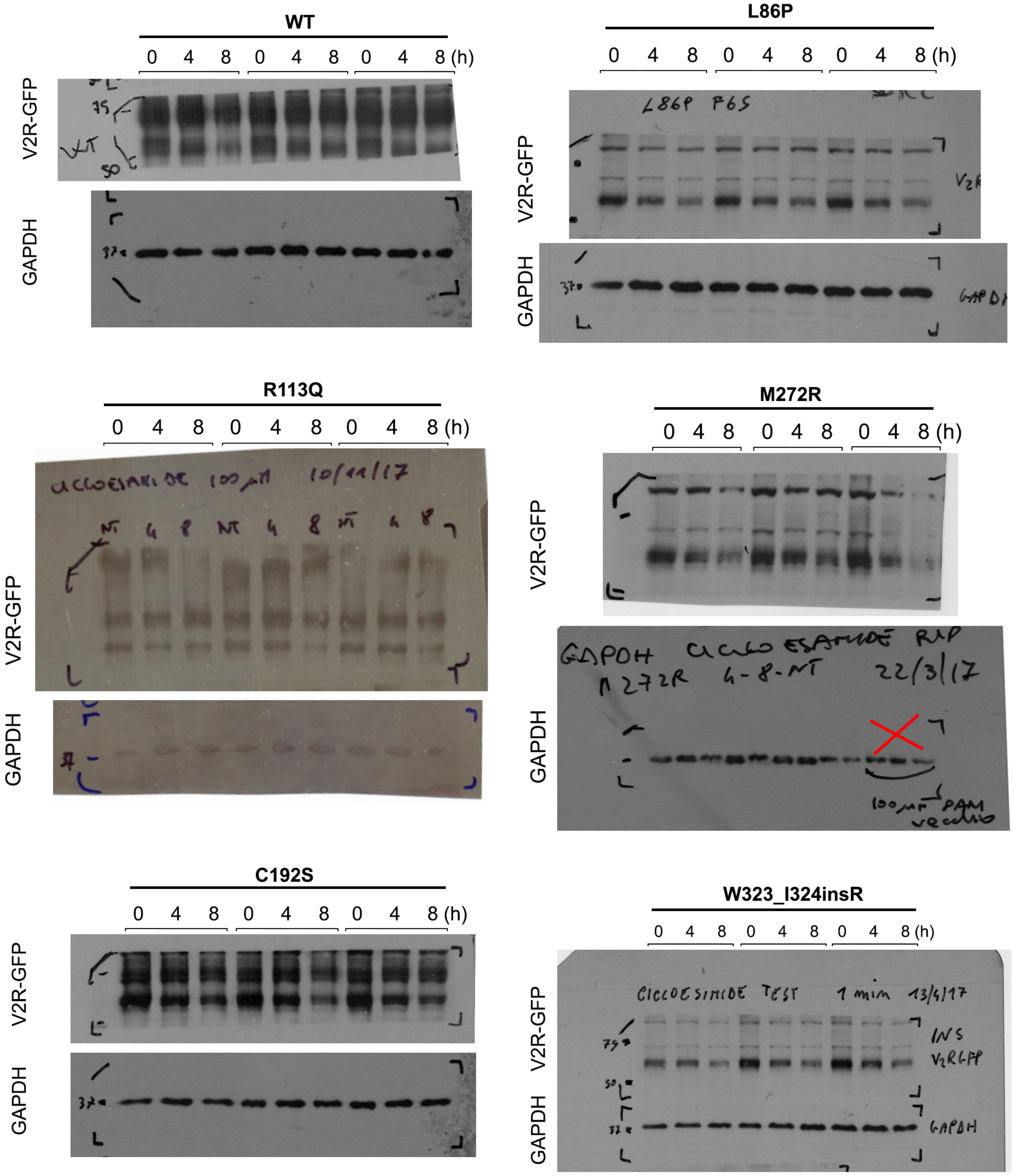

WT
0 4 8 0 4 8 0 4 8 (h)
V2R-GFP
GAPDH
L86P
0 4 8 0 4 8 0 4 8 (h)
V2R-GFP
GAPDH
R113Q
0 4 8 0 4 8 0 4 8 (h)
V2R-GFP
GAPDH
M272R
0 4 8 0 4 8 0 4 8 (h)
V2R-GFP
GAPDH
C192S
0 4 8 0 4 8 0 4 8 (h)
V2R-GFP
GAPDH
W323_I324insR
0 4 8 0 4 8 0 4 8 (h)
V2R-GFP
GAPDH

Fig. 4A, Supplementary Fig S2

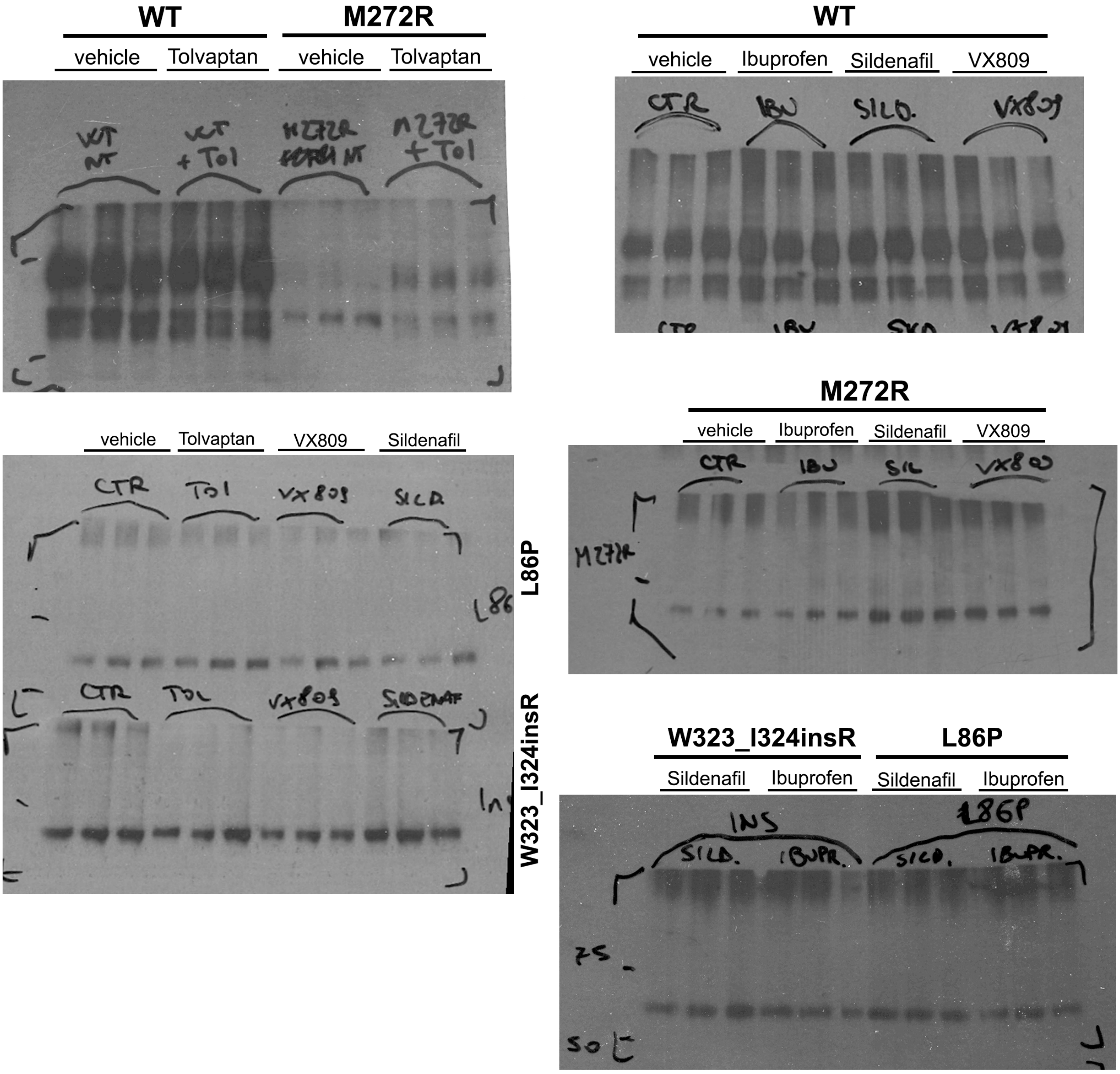

Supplement: Supplementary file 1 — Supplementary Information. [file 41598_2020_73089_MOESM1_ESM.pdf]
